# Discharge preparedness in chronic heart failure: a dyadic qualitative study of patient and clinician perspectives

**DOI:** 10.3389/fcvm.2026.1772485

**Published:** 2026-03-20

**Authors:** Shan Li, Ruonan Yang, Zhaoying Zhu, Wenqian Wang, Siying Ji, Yunying Hou

**Affiliations:** 1 Department of Nursing, The First Affiliated Hospital of Soochow University, Suzhou, China; 2 School of Nursing, Suzhou Medical College of Soochow University, Suzhou, China

**Keywords:** chronic heart failure, discharge preparedness, dyadic analysis, patient-centered care, transitional care

## Abstract

**Background:**

Chronic heart failure (CHF) in China is characterized by high readmission rates, often linked to suboptimal discharge preparedness. Current discharge planning research predominantly relies on unilateral perspectives—focusing either solely on patient compliance or clinician workflows—thereby failing to capture the critical interactional misalignments that compromise care transitions. This study addresses this gap by employing a dyadic lens to expose the latent discrepancies between clinical priorities and patients’ lived realities.

**Methods:**

A descriptive qualitative study was conducted at a tertiary hospital in urban China (November 2024–March 2025). Semi-structured interviews were conducted with 15 CHF patients and 6 cardiovascular clinicians. Data were analyzed using a directed content analysis approach underpinned by Spencer's biomedicalization framework. This theoretical orientation provided a structured methodology to decode the epistemic asymmetries and power dynamics characterizing the discharge process.

**Results:**

Four themes emerged: 1) The Disconnect in Medical Understanding (mechanistic misconceptions vs. educational barriers); 2) Fragile Self-Management in Daily Life (symptom-driven adherence vs. monitoring neglect); 3) Socio-Cultural Conflicts in Care (dietary non-compliance and contextual activity barriers); 4) Systemic Gaps in Support (familial support deficits and healthcare system shortfalls).

**Conclusion:**

Discharge preparation deficiencies reflect bidirectional misalignments between patients’ psychosocially embedded needs and clinicians’ biomedical risk focus. An integrated intervention framework is proposed: 1) Patient-tier: Literacy-adapted education co-construction; 2) Family-tier: Caregiver crisis-response training; 3) System-tier: Digitally-enhanced nurse-led care coordination. Keywords: Chronic heart failure, Discharge planning, Health literacy, Patient-centered care, Transitional care.

## 1 Introduction

Chronic heart failure (HF) imposes a profound global burden, with escalating prevalence due to aging populations and improved cardiac survival rates (1, 2). Despite advances in transitional care, 30-day readmission rates remain alarmingly high (20.9%–25.6%) (3, 4), signaling systemic deficiencies in discharge preparation services (DPS) (5). ESC guidelines emphasize daily weight monitoring as foundational to HF management (6), yet real-world studies reveal critical gaps: ≤45% of patients achieve guideline-recommended adherence frequencies, and fewer than 30% comprehend fluid restriction principles (7, 8).

**Table 1:** Sociodemographic characteristics of CHF patients (*N* = 15).

Variable	N	Frequency (%)
Gender		
Male	8	53.3
Female	7	46.7
Age,year		
Mean (SD)	63.8	-
Range	40–75	-
Educational Background		
College Degree or Above	4	26.7
High School	1	6.7
Junior High School or Below	10	66.7
Employment Status		
Employed	7	46.7
Unemployed	3	20.0
Retired	5	33.3
Marital Status		
Married	12	80.0
Single	3	20.0
Residence		
City	9	60.0
County	6	40.0
Residential arrangement		
Living alone	4	26.7
With spouse	4	26.7
With spouse and children	6	40.0
Other	1	6.7

**Table 2:** Professional characteristics of clinical practitioners (*N* = 6).

Variable	N	Frequency (%)
Age, year		
Mean (SD)	42.0	-
Range	35–49	-
Highest Education		
Bachelor's degree	4	66.7
Master's degree	1	16.7
Doctoral degree	1	16.7
Professional Title		
Associate Senior Rank	4	66.7
Intermediate Rank	1	16.7
Senior Rank	1	16.7
Clinical Experience (years)		
Mean (SD)	22.0	-
Range	12–28	-

**Table 3:** Core themes and subthemes in discharge preparedness (dyadic analysis).

Superordinate Theme	Sub-themes	Examples of Significant Codes
1. The Disconnect in Medical Understanding	Mechanistic Confusion	• Taking medication without knowing rationale • Fragmented views of surgical efficacy
	Educational Barriers	• Lack of standardized educational materials • Need for culturally adapted visual aids
2. Fragile Self-Management in Daily Life	Symptom-Driven Adherence	• Arbitrary and reactive dose adjustments • Inconsistent/reactive weight monitoring • Resuming high-risk activities post-discharge
3. Socio-Cultural Conflicts in Care	Dietary Non-Compliance	• Frequent lapses from low-salt diets • Tradition-based sodium consumption
	Contextual Activity Barriers	• Substituting structured exercise with housework • Social settings triggering behavioral relapse
4. Systemic Gaps in Support	Familial Support Deficits	• Emotional isolation and domestic arguments • Caregiver panic during medical emergencies
	Healthcare System Shortfalls	• Need for guidance with digital monitoring tools • Lack of hospital-to-community transition protocols

Traditional Discharge Preparation Services (DPS) often prioritize standardized education—such as weight monitoring and medication adherence—yet overlook the complex negotiation of roles and power imbalances inherent in clinician-patient interactions (9). Recent trials suggest that while structured education improves knowledge, it fails to consistently reduce readmissions (3, 4). This paradox implies that discharge readiness is not merely a technical skill acquisition but a complex, socially negotiated process involving both the provider and the recipient of care.

However, a critical methodological limitation exists in current literature: studies often isolate patient experiences from provider perspectives, treating them as independent silos. This compartmentalization obscures the interactional dynamics—specifically the concordance (or lack thereof) between clinician instructions and patient interpretation—that shape discharge outcomes. A dyadic perspective is therefore essential to reveal how systemic misalignments between biomedical protocols and patients’ psychosocial contexts contribute to care fragmentation.

To scientifically examine these dynamics, this study applies the biomedicalization framework as a critical analytical lens (10, 11). This framework offers a robust methodological basis for exploring ‘epistemic asymmetry’—the divergence between the clinician's technical focus and the patient's experiential knowledge. By situating our inquiry within this theoretical construct, we aim to: (1) explore the lived experiences of discharge preparedness from interwoven patient and clinician perspectives; and (2) identify the structural disjunctures that hinder effective transitional care in China's hierarchical healthcare context.

## 2 Article type

A qualitative study.

## 3 Methods

### 3.1 Design

This study adopts a descriptive qualitative design (12) with a phenomenological orientation (13). We utilized a directed content analysis approach (14) to explore the phenomenon of discharge preparedness. This method allows for the validation and conceptual extension of Spencer's biomedicalization framework while remaining sensitive to the unique, lived experiences of participants within the Chinese healthcare context.

### 3.2 Participants

All interviews were conducted from November 2024 to March 2025 at the First Affiliated Hospital of Soochow University. The purposive sampling method was employed to investigate the clinical practitioners in the Department of Cardiovascular Medicine of the First Affiliated Hospital of Soochow University and the patients with CHF who were hospitalized. Inclusion criteria for patients are as follows: Age ≥ 18 years; Meeting the diagnostic criteria for CHF as stipulated in the “Chinese Guidelines for Diagnosis and Treatment of Heart Failure 2024” (15); Having a minimum of primary school education; Possessing good language expression and comprehension abilities; Having given informed consent and willingness to participate in the study.

Exclusion criteria are as follows: Complicated with other severe diseases such as tumors, organ failure of vital organs, etc.; History of neurological and psychiatric diseases such as dementia, Parkinson's disease, schizophrenia, bipolar affective disorder, alcohol or other substance dependence.

Inclusion criteria for clinical practitioners are as follows: Working in the cardiovascular department for ≥ 10 years; Having a professional title of intermediate or above; Voluntarily agreeing to participate in this study.

### 3.3 Sample size strategy

Given the specific study aim, the strong theoretical background, and the dense specificity of the dyadic data, a smaller sample of highly experienced clinicians (≥10 years) and diverse patients provided sufficient depth to meet the analytical objectives. Data collection continued until thematic saturation was achieved. Given the specialized expertise required to elucidate systemic barriers in CHF discharge planning, clinician sampling prioritized information power over numerical saturation (16), to capture rich workflow perspectives. Saturation was confirmed when no new thematic insights emerged across three consecutive interviews. Participants were purposefully selected based on their capacity to provide deep insights into care transitions, consistent with Malterud's criteria for sample adequacy in qualitative inquiry (17).

### 3.4 Data collection

Interviews were conducted in a quiet meeting room at the hospital. Qualitative data were collected through semi-structured, face-to-face interviews. All researchers involved in this study were experienced in qualitative research. The interviewer had no prior therapeutic relationship with the participants, minimizing social desirability bias. To develop the semi-structured interview guides, we conducted a comprehensive literature review, including relevant systematic reviews. The final interview protocols used in this study included the following items:

#### 3.4.1 Clinical practitioners were asked

1. What do you consider the key monitoring priorities and health education focus areas for CHF patients during hospitalization?
2. What discharge criteria do you use for CHF patients, and which patient aspects do you prioritize when assessing discharge readiness?
3. How do you provide discharge guidance to CHF patients to facilitate their discharge preparation?
4. Do you consider the current level of discharge readiness among CHF patients to be adequate?
5. What gaps exist in current discharge preparation services for CHF patients, and how might they be addressed?

#### 3.4.2 Patients were asked

1. What was the primary reason for this hospitalization? How has this hospitalization impacted your daily life?
2. How do you feel about discharge? How do you describe your psychological preparedness for life changes after discharge?
3. How would you describe your family members’ understanding of your condition and the support they provide?
4. After discharge, how confident are you in self-managing your condition, monitoring symptoms, and adhering to medications?
5. What challenges might you face after discharge (e.g., physical limitations, psychological stress, financial burden)?
6. What additional support from healthcare providers during hospitalization would improve your post-discharge recovery?
7. How do you perceive the discharge guidance provided, and what improvements would you suggest?

To ensure the richness of data, the interview guide served as a flexible framework rather than a rigid questionnaire. Although the guide included specific topics derived from literature (e.g., symptom monitoring), interviewers used open-ended probes (e.g., “Can you tell me more about that?”, “How did that make you feel?”) to encourage participants to lead the narrative.

To capture real-time discharge readiness, patient interviews were conducted one-on-one within 2 h prior to discharge. Clinician interviews were scheduled during their designated work breaks or pre-arranged availability. Only the interviewee and interviewer were present during sessions. Each interview lasted 30–40 min. Interviewers first introduced themselves to establish rapport. Hydration and breaks were offered during interviews, and breaks were offered if participants experienced fatigue. Audio recordings were transcribed verbatim within 6 h of each interview.

##### 3.4.2.1 Reflexivity and rigor

To ensure methodological rigor and minimize bias, we maintained strict interviewer reflexivity throughout the data collection process. The interviews were conducted by master of nursing candidates who were not involved in the participants’ direct clinical care, thereby establishing a non-hierarchical environment to mitigate social desirability bias. Given the researchers’ clinical backgrounds, we employed phenomenological bracketing (18) to consciously suspend prior medical assumptions and specialized knowledge. This allowed us to remain open to the participants’ authentic lived experiences rather than steering narratives toward clinical expectations. Additionally, interviewers maintained reflexive journals post-interview to document immediate impressions, emotional responses, and potential biases, a practice guided by established reflexivity standards in nursing research (19), which were subsequently discussed with the team to ensure interpretative validity.

### 3.5 Data analysis

Qualitative content analysis guided the analytical process. All audio-recorded interviews with clinical practitioners and patients were transcribed verbatim and cross-verified for accuracy. Research team members independently immersed themselves in the data through repeated reading of transcripts to identify preliminary insights from both perspectives. Meaningful phrases or sentences capturing key experiences (e.g., patient anxieties about symptom management, nurse frustrations with resource constraints) were manually extracted and coded using color annotations and spreadsheets. Analysis followed a hybrid approach integrating inductive coding with deductive directed content analysis (20). Initially, open coding was performed to capture participant-driven themes (phenomenological focus). Subsequently, codes were mapped against Spencer's biomedicalization constructs to identify structural power dynamics (21). This abductive process ensured that findings were grounded in participant data while being theoretically informed. Divergent perspectives were maintained through separate patient/clinician coding matrices before integration. Through constant comparison and abstraction, subcategories were refined into broader themes, explicitly exploring convergences and tensions between clinical practitioners and patient narratives. To enhance trustworthiness, emerging themes were shared with four participating clinical practitioners and five patients for member checking; all confirmed the findings resonated with their experiences. Final thematic consensus was achieved through team deliberation, with coding discrepancies (<5% resolved via transcript re-examination). This process yielded four core themes reflecting the complexities of discharge preparation in chronic heart failure care.

### 3.6 Ethical considerations

Approved by Ethics Committee of Soochow University (No. SUDA20241209H11). Written informed consent included publication of anonymized quotes; data were anonymized and stored securely.

## 4 Results

Through rigorous analysis of semi-structured interviews with 15 chronic heart failure (CHF) patients and 6 cardiovascular clinical practitioners, four core themes and eight sub-themes emerged, capturing critical dimensions of discharge preparedness from dual perspectives. Verbatim quotes (labeled as P1–P15 for patients, HCP1–HCP6 for clinical practitioners) illustrate key findings. The demographic and professional characteristics of the 15 CHF patients and 6 clinical practitioners are presented in Tables 1 and 2, respectively. An overview of the four core themes and sub-themes is provided in Table 3.

### 4.1 Theme 1: the disconnect in medical understanding

Patients exhibited significant gaps in understanding CHF pathophysiology and self-management principles, while clinical practitioners emphasized systemic educational barriers. This disconnect exemplifies the epistemic asymmetry described in the biomedicalization framework, where the clinician's focus on technoscientific accuracy inadvertently marginalizes the patient's experiential understanding of their condition.

Mechanistic Confusion: Patients often viewed treatment through a fragmented lens. Low-literacy patients expressed confusion about treatment rationale (*P2: “I just take pills without knowing what they're for”*), reflecting disempowerment within hierarchical knowledge transmission. Even educated patients demonstrated fragmented knowledge (*P4: “Surgery fixes blocked vessels, so why worry?”*).

Educational Barriers: Clinical practitioners identified inconsistent patient education as a root cause (*HCP4: “Standardized materials are lacking—we repeat basics daily”*). Clinical practitioners stressed the need for culturally adapted visual aids to explain fluid overload and medication actions.

### 4.2 Theme 2: Fragile self-management in daily life

Discrepancies emerged between patients’ self-reported behaviors and clinical practitioners’ observations of adherence. The contrast between patients’ symptom-driven adjustments and clinicians’ strict adherence expectations highlights the tension between standardized biomedical protocols and the fluid, embodied reality of living with chronic illness.

Symptom-Driven Adherence: Adherence was often reactive rather than proactive. Patients described arbitrary dose adjustments (*P10: “I stop diuretics if swelling improves”*), contrasting with clinical practitioners’ reports of avoidable readmissions due to self-altered regimens (*HCP2: “They skip vital drugs like ACE inhibitors when asymptomatic”*). While 11/15 patients acknowledged weight monitoring importance, only 3 practiced daily tracking (*P7: “I only weigh if clothes feel tight”*). Clinical practitioners highlighted poor infection prevention awareness (*HCP5: “Patients resume high-risk activities post-discharge”*).

### 4.3 Theme 3: socio-cultural conflicts in care

Patients recognized ideal behaviors but faced social cultural environment barriers to implementation. These conflicts illustrate the limitations of biomedical surveillance when it intrudes into the private sphere. The persistence of cultural habits represents a form of resistance against the technoscientific transformation of daily life, where patients struggle to reconcile their social identities with the imperative to become a ‘biomedicalized subject’ governed by risk metrics.

Dietary Non-Compliance: Dietary intentions frequently conflicted with culturally embedded practices. All patients endorsed “low-salt diets,” yet 9/15 admitted frequent lapses (*P9: “Avoiding fatty meats complies with advice, but pickled vegetables are tradition—I didn't know their sodium risk.”*).

Contextual Activity Barriers: Despite receiving exercise plans, 12 patients were constrained by their social environment and instead used daily chores and tasks to replace structured exercise. (*P13: “Doing housework replaces exercising”*). Clinical practitioners noted environmental triggers (*HCP3: “Smoking relapse occurs in social settings—we need community interventions”*).

### 4.4 Theme 4: systemic gaps in support

Both groups identified critical deficiencies in familial and institutional support. This systemic disconnection highlights a critical flaw in stratified biomedicalization: the devolution of clinical responsibility to the domestic sphere is not matched by adequate technoscientific infrastructure. Consequently, vulnerable patients experience a form of structural abandonment, where the continuity of care is fractured by institutional barriers.

Familial Support Deficits: Patients reported emotional isolation (*P1: “Arguments with my wife landed me back in hospital”*) and inadequate crisis support (*P13: “My spouse panics during emergencies”*). Clinical practitioners emphasized caregiver training gaps (*HCP6: “Families can't recognize early decompensation”*).

Healthcare System Shortfalls: Patients desired post-discharge digital tools (*P9: “Smartwatches help, but I need guidance”*), while clinical practitioners advocated for structured transitions (*HCP1: “We lack protocols linking hospital to community care”*).

## 5 Discussion

This dual-perspective study demonstrates that suboptimal discharge preparedness among CHF patients stems from interdependent patient-level cognitive-behavioral gaps, relational support deficiencies, and systemic fragmentation. Four thematic domains elucidate this complexity:

### 5.1 Reinterpreting knowledge gaps: from compliance to co-construction

Our findings reveal a fundamental disconnect: while clinicians focused on the transmission of biomedical protocols, patients often struggled to apply this information to their daily lives due to mechanistic misconceptions (Theme 1). For instance, patients like P2 viewed medication intake as a passive task without understanding the rationale, a finding that aligns with the foundational tenets of biomedicalization (10) and Spencer's concept of ‘epistemic asymmetry’ (11). This theoretical lens suggests that when discharge education is treated as a unidirectional delivery of technical knowledge, it inadvertently erodes patient agency. Instead of mere non-compliance, patients’ substitution of protocols with vernacular practices (e.g., P9's dietary choices) represents a form of adaptation to this rigid biomedical dominance. Therefore, discharge preparedness should not be viewed solely as a technical skill transfer but as a negotiated process. This implies that interventions must move beyond standard education to knowledge co-construction, where clinicians actively solicit and integrate patients’ cultural and lifestyle contexts into care plans (22), a strategy shown to enhance self-efficacy in similar chronic disease populations (23).

### 5.2 The double-edged sword of familial dynamics

Contrary to the assumption that family presence guarantees support, our study highlights that relational conflicts often undermine discharge readiness (Theme 4). Participants reported that family stressors, such as arguments or caregiver panic during emergencies, directly exacerbated their vulnerability (e.g., P1, P13). Within the Chinese context, these dynamics are complicated by Confucian familial hierarchies, where caregivers often hold decision-making power yet may lack the medical literacy to manage decompensation events effectively (24). Unlike collectivist models in other contexts that successfully buffer stress through shared kinship (25), our findings suggest that without specific training, the heavy burden on untrained caregivers can lead to friction. This underscores the necessity for dyadic interventions that treat the patient-caregiver unit as a whole. Future nursing strategies should likely include conflict resolution and crisis simulation training for families (26), tailored to respect local hierarchical norms while empowering caregivers with practical competence.

### 5.3 Navigating the digital divide in transitional care

While digital tools offer a promising avenue for post-discharge monitoring, our results expose a significant ‘digital divide’ that mirrors socioeconomic disparities (Themes 3 & 4). We observed a sharp contrast between younger, tech-savvy patients who utilized wearables and older or rural participants who felt alienated by complex interfaces. This aligns with broader evidence that technological accessibility does not equate to usability for vulnerable groups (27). The reliance on ‘Internet + Healthcare’ initiatives (27) runs the risk of exacerbating systemic inequities if the specific barriers identified in our study—such as low eHealth literacy and lack of human guidance—are not addressed. Consequently, digital implementation in CHF care must be equity-centric. This suggests that high-tech solutions should be complemented by ‘high-touch’ nurse-led navigation to ensure that patients unable to engage with digital platforms are not left behind in the transition from hospital to home.

### 5.4 Addressing systemic discontinuities through nurse-led integration

Finally, the systemic discontinuities identified by both clinicians and patients (Theme 4) point to a structural gap in the continuity of care. The lack of standardized handoffs between tertiary hospitals and community providers resulted in patients feeling abandoned post-discharge. This fragmentation validates the need for a nurse-led transitional care model that bridges these silos. As the primary coordinators of discharge planning, nurses are uniquely positioned to orchestrate this continuity (28). Our findings support the development of protocols where discharge preparation involves direct linkage to community resources, ensuring that the ’safety net’ extends beyond the hospital walls.

## 6 Limitations

Several limitations warrant consideration when interpreting these findings. First, the study was conducted at a single tertiary center in urban China; thus, the experiences may not fully reflect those of patients in rural or primary care settings, where resource constraints differ. Second, while the dyadic design is a strength, the sample of clinicians was relatively small (*n* = 6). Although information power was prioritized and thematic saturation was achieved, the findings regarding clinician workflows may vary across different hospital cultures. Third, the cross-sectional nature of the interviews captures discharge readiness at a specific moment in time; a longitudinal design could better illuminate how these perceptions evolve post-discharge. Finally, acknowledging that the researcher serves as the primary instrument in qualitative inquiry, our analysis inevitably reflects a degree of interpretative subjectivity. However, we mitigated this potential bias through sustained reflexivity, including the bracketing of clinical preconceptions and rigorous member checking, to ensure the findings authentically represent the participants’ perspectives.

## 7 Clinical implications

This study delineates three evidence-informed clinical priorities to optimize discharge readiness. First, implementing literacy-stratified education is paramount, utilizing visual medication schematics and teach-back verification for patients with limited health literacy, while employing decision-balance frameworks for educated but non-adherent individuals. Concurrently, integrating family-centered interventions must address relational dynamics through decompensation recognition drills for caregivers and conflict de-escalation skills training to mitigate familial stressors implicated in readmissions. Critically, strengthening transitional infrastructure through EHR-enabled nurse coordination with community providers which aligns with China's hierarchical medical system reform objectives (1, 29). These multilayered strategies—targeting patient cognition, relational support systems, and structural fragmentation—collectively substantiate a patient-centered framework for reducing preventable readmissions.

## 8 Conclusions

This qualitative analysis elucidates critical barriers and facilitators in discharge preparedness for CHF patients through clinician-patient dyadic perspectives. Four themes—the disconnect in medical understanding, fragile self-management in daily life, socio-cultural conflicts in care, and systemic gaps in support—reveal synergistic challenges: patients struggle with knowledge application amid psychosocial barriers, while clinical practitioners face systemic gaps in care continuity.

A tiered intervention framework reconciling patient-centered priorities (contextualized self-management support) with clinical imperatives (biomedical risk mitigation) is proposed, comprising: (1) literacy-adapted education co-construction; (2) caregiver competency development; (3) digitally-enhanced nurse-led care coordination.

## Data Availability

The original contributions presented in the study are included in the article/Supplementary Material, further inquiries can be directed to the corresponding author.
